# Optogenetics: Keep Interpretations Light

**DOI:** 10.1523/ENEURO.0091-20.2020

**Published:** 2020-04-16

**Authors:** Christophe Bernard

New technologies always bring (over)excitement, because they allow us to perform “so far only dreamed of” experiments. It is only later that one discovers the caveats. Many of us remember the excitement brought about by the ability to knock out genes. The first papers strongly relied on the belief that if knocking out gene X prevents observation Y from happening, it shows causality, i.e., the function of X is to control Y directly. We now know that we must be more careful with data interpretation. For example, knocking out X may result in a misconstruction of the brain that will lead to an alteration in Y (i.e., X does not control Y directly). Such explanations are particularly important for the interpretation of preclinical studies (Marguet et al., 2015).

Optogenetics is not an exception to the rule. When using light to control cells, transcription, etc., we believe that if we activate cell X with light and observe Y, then Y is due to the activity of X. Let’s review some potential known and unknown confounding factors.

## Brain Dynamics

At rest, brain activity displays complex dynamic patterns that can be seen using fMRI, EEG, or mesoscopic Ca^2+^ imaging. These patterns reflect the activity of all cells, even specific cells (Xiao et al., 2017), and such activity patterns may, in turn, affect the future activity of all cells. Neurons are embedded in circuits in constant interaction with each other. If we force some cells to be active (or inactive), whole-brain dynamics may be affected, which may explain observation Y. Feedforward and feedback loops may be activated, resulting in a complex effect. For example, the photoactivation of some parvalbumin cells containing red-shifted channelrhodopsin results in an unexpected decrease in their activity (Mahrach et al., 2020). In the latter case, Y would be the result of inhibition, not activation, of X cells. Therefore, network effects need to be considered for a proper interpretation of the results.

## Cell Dynamics

The ultimate effect of opsin activation is not always clear. For example, some cells may quickly enter a depolarization block after shining light on them. Hence, Y could be due to an inhibitory effect, as the cells cannot fire when in a depolarization block. Conversely, hyperpolarizing cells with light may indeed prevent their firing. However, as soon as the light is off, all “inhibited” cells may produce a hugely synchronized firing response due to postinhibitory rebound, for example, if the cells express the h current. Postinhibitory rebound firing occurs *in vivo* in physiological conditions (Adhikari et al., 2012). In that case, Y would result from the highly synchronized firing of X cells, not their inhibition.

## Xenoproteins

We can introduce xenoproteins into cells to control them. Channelrhodopsins are large proteins with, potentially, many interacting sites. Proteins can have multiple functions. Can channelrhodopsins have a direct cytosolic action? If they bind to the cytoskeleton, do they prevent the normal insertion of native proteins, thus perturbing normal processes? If the biology of the cell is modified, are its functions also modified? And if cellular functions are modified, cell X will not be in its “normal” state, and Y will be the result of the activation of an “abnormal” state. Many of these issues remain to be investigated.

## Stay Away from the Light

Light is made of photons, hence energy plus matter. Provide enough energy to atoms, and various things will happen, including perturbing hydrogen bonds. This is the mechanism underlying the opening of channelrhodopsin (Volkov et al., 2017). What if light is also perturbing hydrogen bonds in native proteins or complexes of proteins? Going back to physics, shining light in the brain will generate a lot of photons. The photons may be absorbed by atoms, adding energy to their orbital electrons, and thus kinetic energy to the nucleus of the atoms, ultimately resulting in a rise in temperature. Since molecular interactions, enzymatic reactions, conformational changes of molecules, etc. are very sensitive to changes in temperature, the mere action of shining light may alter cell biology/function. Among other things, photons change the biophysical properties of NMDA receptors (Leszkiewicz et al., 2000) and alter gene expression (Cheng et al., 2016; Volkov et al., 2017; Tyssowski and Gray, 2019; Duke et al., 2020). In these conditions, even if activation of cell X leads to observation Y, while light only (X does not express opsins) does not induce Y, it does not rule out the possibility that Y is the result of the activation of X plus the photon effect (i.e., to obtain Y, you need both X activation and the photon effect).

## Virus: Malware for Your Software

Optogenetics can make use of viruses, which may themselves trigger morphologic and functional modifications (Miyashita et al., 2013; Jackman et al., 2014), including ablating neurogenesis (Johnston et al., 2020). Arguably, Y can only be observed in the context of a circuit modified by the virus, i.e., it would never be observed in physiological conditions when X is active.

## Perturbation

The photon effect, the use of viruses, and the expression of xenoproteins are strong perturbators of neural systems. Let us now consider two other important confounding factors. Optogenetic control usually makes use of an optic fiber that has to go through several brain regions before reaching the target area, sometimes destroying large pieces of tissue on the way (triggering bleeding, inflammatory responses, etc.). Therefore, fiber insertion alone may alter whole-brain dynamics and perhaps the “normal” effect of cell X activation. Hence, like virus injection, a neural system may be in a pathologic state. Finally, many experimental protocols require single housing of animals, if only to protect the equipment carried by the animal. Rodents are social animals and isolating them, even in an enriched environment, is extremely stressful to them (Manouze et al., 2019). If social interaction is not provided, how can we rule out that Y is only observed under stress conditions when X is activated?

In conclusion, as for an experimental protocol that requires perturbing a neural system, one always needs to be aware of the limitations of the techniques and what it means for data interpretation. The observations are correct, but it is their interpretation that is challenging. Optogenetics (and chemogenetics) are amazing techniques that have enabled us to make major leaps forward. However, one must be very careful when claiming causality. The observation of Y is indeed the result of activation of X but with many other possible steps and pathways in-between.

I also encourage you to visit the tutorial on the caveats and limitations of optogenetics hosted on the Society for Neuroscience’s member platform, Neuronline (https://neuronline.sfn.org/training/module-8-caveats-and-limitations-of-optogenetics).
